# Chemical characterization and i*n vitro* immunomodulatory effects of different extracts of moss *Hedwigia ciliata* (Hedw.) P. Beauv. from the Vršačke Planine Mts., Serbia

**DOI:** 10.1371/journal.pone.0246810

**Published:** 2021-02-11

**Authors:** Marija R. Mandić, Mariana M. Oalđe, Tanja M. Lunić, Aneta D. Sabovljević, Marko S. Sabovljević, Uroš M. Gašić, Sonja N. Duletić-Laušević, Bojan Dj. Božić, Biljana Dj. Božić Nedeljković

**Affiliations:** 1 Faculty of Biology, Institute of Physiology and Biochemistry “Ivan Djaja”, University of Belgrade, Belgrade, Serbia; 2 Faculty of Biology, Institute of Botany and Botanical Garden "Jevremovac", University of Belgrade, Belgrade, Serbia; 3 Department of Plant Physiology, Institute for Biological Research “Sinisa Stankovic”, National Institute of Republic of Serbia, University of Belgrade, Belgrade, Serbia; National University of Kaohsiung, TAIWAN

## Abstract

Bioactive compounds from natural sources are of great importance because of their potential pharmacological activity and tremendous structural diversity. In this study, the chemical composition of different moss extracts of *Hedwigia ciliata* P. Beauv. have been examined, as well as their antioxidant, antineurodegenerative/anti-neuroinflammatory, antidiabetic, and antiproliferative potential. The extracts were prepared by Soxhlet extractor using solvents of different polarity. Chemical characterization of the extracts revealed the presence of phenolics and flavonoid compounds, together with triterpenoids as secondary metabolites of high biological activity. Significant antioxidant properties of all the extracts were exhibited using the β-carotene assay. The highest activities were found for water:ethanol extract (with the highest inhibition rate of 96%), but also significant inhibition was measured for ethanol and ethyl acetate extracts (80% and 70%, respectively). Confirmation of biocompatibility of investigated moss extracts has been performed using normal human fibroblast cell line, MRC-5. The *H*. *ciliata* extracts exhibited significant antiproliferative activity (~ 50%) against the MDA-MB-231 (human breast adenocarcinoma cell line), which has not previously been reported elsewhere. The Griess assay confirmed the potential anti-neuroinflammatory activity of the extracts, as significant effects in reducing NO production by LPS-stimulated BV2 (normal murine microglia cell line) was observed. This data is in line with noted antineurodegenerative potential measured by the inhibition of acetylcholinesterase (with the highest inhibition rate of 60% for ethyl acetate extract) and tyrosinase (with the highest inhibition rate of 70% for ethanol extract). Additionally, the *H*. *ciliata* extracts exhibited significant antidiabetic effect mediated by α-glucosidase inhibition (with the highest inhibition rate of 80% for ethyl acetate extract). The obtained data suggest the presence of immunomodulatory effects of the moss extracts *in vitro*, which allows the design of new experiments aimed at detecting and characterizing bioactive compounds of the extracts and additionally elucidate detailed mechanisms of their effects.

## Introduction

Numerous studies have indicated the presence of immunomodulatory molecules in various plant extracts, with some highlighting the potential use of phenolics, flavonoids, and terpenoids as immunomodulatory agents [1,2]. Immunomodulators can successfully offer methods to enhance or decrease immune response and prevent various infections, tumors, different inflammatory disorders, and stress-related diseases [3]. As there is a growing demand for new active compounds and design of new drugs, mosses provide the opportunity to discover new antimicrobial and antitumor therapeutic agents [4].

Bryophytes represent a large group of plants that are widespread in almost every part of the world. They are divided into three phyla–mosses, liverworts, and hornworts. Mosses grow on various types of substrates such as soil, stones, rocks, trees, rotting trunks, roofs, in lakes and rivers [5]. Their distribution depends on abiotic factors as well as geo-historical events. There are evidences that the first species of mosses appeared 472 million years ago, making them one of the oldest land plants on the planet [5,6].

Although mosses are not used in human nutrition, they have been used in traditional medicine since ancient times, especially in China, Europe, North America and India [5]. Due to their antimicrobial properties, mosses were used to treat a variety of pathological conditions including bronchitis, skin infections, as well as for the healing of wounds and burns [5,7]. Despite their beneficial effects, the exact mechanisms underlying the moss bioactivity have not been sufficiently studied. However, they have become the subject of research in recent studies, in order to investigate their potential immunomodulatory effects and use for the development of new therapeutics [8]. In the present study, the chemical and biological properties of moss *Hedwigia ciliata* were examined. It belongs to acrocarp perennial mosses, which achieve ca. 2.5 cm in height and up to 30 cm in width. Individual stalks are red-brown in color, with leaves of moderate density rising. The leaves are 1.5–2 mm long, green or yellow-green in color, with sharp white tips. They usually grow in sunny, acidic rocky areas and are widespread in all continents except Antarctica [9]. Previous studies have reported antioxidant, antimicrobial and antitumor activities of *H*. *ciliata* extracts [10]. Mosses consist of the same basic organic substances as other plants, differing in the percentage of certain components. The major part consists of hemicellulose and pectin (30–60%), followed by cellulose (15–25%), proteins (5–10%), polyphenols (5–10%), and inorganic substances (3–10%) [11]. Literature data indicate that different extraction and analytical methods were applied for examination of chemical profile of mosses. Due to the different polarity of the active substances, the choice of extraction method and solvent is of particular importance, with water, methanol, ethanol, ethyl acetate, hexane, chloroform, and dichloromethane mainly used [11].

Most research has focused on the examination of secondary metabolites that have shown high biological activity. Secondary metabolites represent a large group of biologically active compounds that are synthesized as a plant defense mechanism against various microorganisms, insects, herbivores, and different extreme of environmental conditions, as well as for UV protection [11,12]. Some of the currently identified secondary moss metabolites belong to a group of compounds such as phenolic compounds, flavonoids, and terpenoids [4,13] which have been shown to have antioxidant, anti-inflammatory, antitumor, and antibacterial effects [1,4,14,15]. Flavonoid glycosides such as apigenin and luteolinhave already been confirmed in *H*. *ciliata* [16–18].

This study aimed to investigate the potential bioactive effects of various extracts of *H*. *ciliata*, in *in vitro* conditions. The chemical characterization of extracts, as well as the examination of their therapeutic effect, may indicate the mechanisms underlying the immunomodulation processes. By knowing these processes, we gain new insights that can be applied to treat some pathological conditions.

## Materials and methods

### Reagents and standards

Acarbose, acetylcholinesterase (from *Electrophorus electricus*), acetylcholine iodide, ascorbic acid, α-amylase, α-glucosidase (from *Saccharomyces cerevisiae*) type I, aluminum chloride (AlCl_**3**_), BHA (2-tert-butyl-4-hydroxyanisole), BHT (3,5-di-tert-butyl-4-hydroxytoluene), β-carotene, caffeic acid, coumarin, disodium hydrogen phosphate dodecahydrate, DMSO (dimethyl sulfoxide), DPPH (2,2-diphenyl-1-picrylhydrazyl), DTNB (5,5’-dithio-bis(2-nitrobenzoic acid)), Folin-Ciocalteu reagent, galantamine, gallic acid, iron(III) chloride (FeCl_**3**_), L-DOPA (3,4-dihydroxy-L-phenylalanine), Lugol’s solution, MTT (3-(4,5-dimethylthiazol-2-yl)-2,5-diphenyltetrazolium bromid), pNPG (4-nitrophenyl β-D-glucopyranoside), potassium dihydrogen phosphate, phosphoric acid, quercetin, sodium nitrite (NaNO_**2**_), sulfanilamide, sulfanilic acid, sodium acetate (CH_**3**_COONa), sodium carbonate anhydrous (Na_**2**_CO_**3**_), sodium bicarbonate (NaHCO_**3**_), sodium chloride (NaCl), sodium phosphate monobasic dihydrate, sodium hydrogen phosphate, sodium dihydrogen phosphate, Trizma base, tyrosinase (from *Agaricusbisporus*), ursolic acid, vanillin and kojic acid were purchased from Sigma-Aldrich, St. Louis, MO, USA. Aluminum nitrate nonahydrate (Al(NO_**3**_)_**3**_ × 9H_**2**_O) and potassium acetate (CH_**3**_COOK) were obtained from Carlo Erba Reagents, Barcelona, Spain. Chloroform, ethanol, glacial acetic acid and hydrochloric acid were obtained from Zorka Pharma, Šabac, Serbia. Dipotassiumphosphate, methanol and perchloric acid were purchased from VWR, Radnor, PA, USA. Linoleic acid and Tween 40 were purchased from Acros Organics, Geel, Belgium. DMEM (Dulbecco’s Modified Eagle Medium), FBS (Fetal Bovine Serum) and PBS (Phosphate-Buffered Saline) were obtained from GIBCO, Invitrogen, Carlsbad, CA, USA. NBT (Nitro Blue Tetrazolium) was obtained from SERVA, Heidelberg, Germany. Lead acetate trihydrate (Pb(C_**2**_H_**3**_O_**2**_)_**2**_ × 3(H_**2**_O)), potassium ferricyanide (III) and trichloroacetic acid were obtained from Superlab, Belgrade, Serbia. Sodium molybdate (Na_**2**_MoO_**4**_ × 2H_**2**_O) was obtained from Dispo-chem, Romsey, UK. Sodium hydroxide (NaOH) was purchased from NRK inženjering, Belgrade, Serbia. Starch solution (1%) was purchased from Carl Roth, Karlsruhe, Germany.

### Plant material

Specimens of moss *H*. *ciliata* (spring 2019 aspect) were collectedin Vršačke Planine Mts. (Serbia) (N45° 7’ 41.5488", E21° 19’ 47.8014", 370 m a.s.l.) by random sampling from the siliceous rock outcrops at the forest openings (leg./det. M. S. Sabovljevic and A. D. Sabovljevic, 11.05.2019; voucher BEOU bryo collection s/n).Permission for the plants collection for research purposes within the territory of Serbia is issued by the Ministry of Environment of the Republic of Serbia (permission number 353-01-572/2018-04). The moss species used in this study is not protected in Serbia nor under threat, red-listed and nationally or internationally of any conservation issue [19,20]. Mosses were collected in paper bags and kept on room temperature. The room-dried and cleaned material (i.e. green tips with no older parts and substrate remnants) was then lyophilized, being ready for extraction.

### Preparation of the extracts

The samples were dried by lyophilisation method to constant weight, and then powdered and weighed (Mass 1). According to previous studies [4,14] moss chemical composition have shown a wide range of various polar and non-polar active compounds, thus solvent properties will significantly be influenced on the chemical composition of their extract. Therefore, three typesof different polarity solvents and corresponding mixture have been used for moss extraction: 96% ethanol, water:ethanol (50:50, vol%), and ethyl acetate. Optimization of the extraction process was accomplished by monitoring the change of absorption maximum at 673 nm wavelength of the ethanol extract, every two hours using UV spectrophotometry. The absorption value reached the peak after 10 hours of the extraction, thus it was applied for other solvents as the optimum time for the extraction. After optimization of the conditions, the extraction was completed in Soxhlet for 10 hours. The resulting extracts E1 (96% ethanol), E2 (water:ethanol– 50:50, vol%), and E3 (ethyl acetate) were concentrated by vacuum evaporation (Buchi R-210 Rotavapor System) and evaporated to dryness (Mass 2). The extraction yield of the extracts decreased in the following order: E2>E1>E3 (Table 1).

**Table 1:** Extraction yield of the examined extracts.

Extracts	Solvent	Mass 1 (g)	Mass 2 (g)	Yield (%)
**E1**	96% ethanol	10.073	0.6036	6.0
**E2**	50:50 water:ethanol	10.172	1.2316	12.1
**E3**	Ethyl acetate	9.992	0.1970	2.0

**Fig 1:**
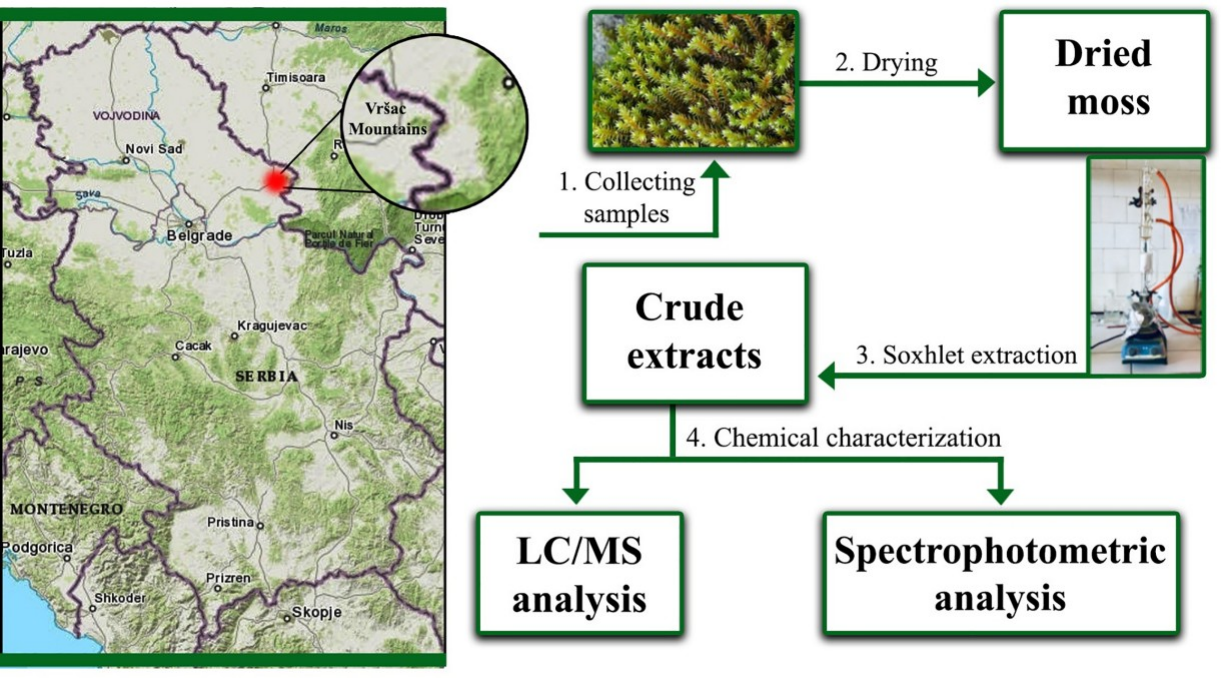
Location of the investigated area (Vršačke Planine Mts., Nature Park, Serbia) and illustrated steps of the preparation and chemical characterization of the *Hedwigia ciliata* extracts. Downloaded and modified from http://viewer.nationalmap.gov/viewer/.

The moss collection and preparation for extraction process, as well as isolation and chemical characterization of the extracts are presented in the Fig 1.

### Characterization of bioactive molecules in extracts

#### Determination of total phenol content

The total content of phenolic compounds was determined using the Folin–Ciocalteau method [21]. In each well on microtitration plate, 100 μL of 10% Folin–Ciocalteu reagent solution was added to 20 μL of each extract (1 mg mL^**-1**^). After incubation (six minutes at room temperature), 80 μL of 7.5% sodium carbonate solution was added. The control contained 100% ethanol instead of the sample. Gallic acid dissolved in distilled water at concentrations of 0.005–0.200 mg mL^**-1**^ was used to construct the calibration curve. After incubation (120 minutes in the dark at room temperature), the absorbance was read at 740 nm using a microtiter plate reader (Multiskan Sky Thermo Scientific, Finland). The total phenol content of the samples was calculated from the calibration curve equation and is presented as gallic acid equivalents, as mg GAE per g dry extract.

#### Determination of total phenolic acid content

Total phenolic acid content was determined according to the modified procedure [22]. In each well on microtiter plate, 20 μL of Arnow reagent (10% w/v sodium molybdate and 10% w/v sodium nitrite, 20 μL 0.1 mol L^**-1**^ hydrochloric acid and 20 μL 1 mol L^**-1**^sodium hydroxide were added to 10 μL of each extract (1 mg mL^**-1**^). Afterwards, another 100 μL of distilled water was added. The control contained 100% ethanol instead of the sample. To construct the calibration curve, caffeic acid dissolved in 50% ethanol at concentrations of 0.0078–1 mg mL^**-1**^ was used. Immediately after all components were added, the absorbance was read at 490 nm using a microtiter plate reader (Multiskan Sky Thermo Scientific, Finland). The total phenolic acid content of the samples was calculated from the calibration curve equation and represented ascaffeic acid equivalents, as mg CAE per g dry extract.

#### Determination of total flavonoid content

The procedure [23] was used to determine the total flavonoid content. In each well on microtitern plate, 205 μL of 80% ethanol, 5 μL of 10% aluminum nitrate nonahydrate, and 5 μL of 1 mol L^**-1**^potassium acetate solution were added to 50 μL of each extract (1 mg mL^**-1**^). The control contained 100% ethanol instead of the sample. Quercetin, dissolved in 96% ethanol at concentrations of 0.005–0.200 mg mL^**-1**^ was used to construct the calibration curve. The absorbance was read after incubation (40 minutes at room temperature) at 415 nm using a microtiter plate reader (Multiscan Sky Thermo Scientific, Finland). The flavonoid content is calculated from the equation of the calibration curve and is expressed as quercetin equivalents, as mg QE per g of dry extract.

#### Determination of total flavonol content

The total flavonol content was determined according to the procedure [22]. In each well on microtiter plate, 40 μL of methanolic solution of aluminium chloride (AlCl_**3**_) (20 mg mL^**-1**^) and 120 μL of methanolic sodium acetate solution (50 mg mL^**-1**^) were added to 40 μL of each extract (1 mg mL^**-1**^). The control contained 100% methanol instead of the sample. Quercetin dissolved in 100% methanol at concentrations of 0.0078–1 mg mL^**-1**^ was used to construct the calibration curve instead of the extract. The absorbance was read at 440 nm after incubation (150 minutes) using a microtiter plate reader (Multiskan Sky Thermo Scientific, Finland). The total flavonol content of the samples was calculated from the calibration curve equation and represented as quercetin equivalents, as mg QE per g of dry extract.

#### Determination of total triterpenoid content

The total triterpenoid content was determined according to the following procedure [24]. In each well on microtiter plate, 10 μL of extract (10 mg mL^**-1**^) was added, followed by the addition of 15 μL of vanillin-glacial acid solution (5% v/v) and 50 μL of perchloric acid solution. The reaction mixture was incubated for 45 minutes at 60°C. After cooling to room temperature, 225 μL of glacial acetic acid was added. The control contained 100% methanol (0.005–1 mg mL^**-1**^) instead of the sample. Absorbance was measured at 548 nm, using a Multiskan Sky Thermo Scientific microtiter plate reader, Finland. Ursolic acid dissolved in 100% methanol was used to construct the calibration curve. All measurements were repeated three times. The total triterpenoid content of the samples was calculated from the calibration curve and represented as equivalents of ursolic acids, as mg UAE per g of the extract.

#### Determination of total coumarin content

Determination of the total content of coumarin was performed according to the procedure [25] with modifications. In each well on microtiter plate, 2 μL of extract (10 mg mL^**-1**^), 8 μL of distilled water, and 2 μL of lead acetate trihydrate solution (5% w/v) was added. Another 28 μL of distilled water was added to each well, followed by 160 μL of 0.1 mol L^**-1**^ hydrochloric acid. The reaction mixture was incubated for 30 minutes at room temperature. The control contained 100% methanol instead of the sample. Absorbance was measured at 320 nm using Multiskan Sky Thermo Scientific microtiter plate reader, Finland. Coumarin dissolved in methanol (0.005–1 mg mL^**-1**^) was used to construct the calibration curve. All measurements were repeated three times. The total coumarin content of the samples was calculated from the calibration curve equation and is presented as coumarin equivalents, as mg CE per g of the dry extract.

#### Liquid chromatography-mass spectrometry

Separation of compounds of interest was performed using a Dionex Ultimate 3000 UHPLC system equipped with a diode array detector (DAD) that was connected to TSQ Quantum Access Max triple-quadrupole mass spectrometer equipped with heated electrospray ionization probe (HESI-II, Thermo Fisher Scientific, Bremen, Germany) in negative ionization mode.

A Syncronis C18 column (100 × 2.1 mm, 1.7 μm particle size) at 40°C was used for compounds separation: Flow rate was set of 0.3 mL/min and the mobile phase was consisted of (A) water + 0.1% formic acid and (B) acetonitrile. Linear gradient program was used as follows: 0.0–1.0 min 5% B, 1.0–14.0 min from 5% to 95% (B), 14.0–14.1 min from 95% to 5% (B), and 5% (B) for six minutes.

Parameters of the ion source and the other MS data necessary for quantification were as previously described in the literature [26]. The MS data were acquired in negative mode in *m/z* range from 100 to 1000. Full scanning (FS), product ion scanning (PIS) and neutral loss scanning (NLS) were conducted for the qualitative analysis. Collision-induced fragmentation experiments were performed with collision energy set to 30 eV. A selected reaction monitoring (SRM) experiment for quantitative analysis was performed using two MS^**2**^ fragments for each compound, which were previously defined as dominant in PIS experiments.

A 10 g/L stock solution of a mixture of all standards was prepared in methanol. Dilution of the stock solution with mobile phase (acetonitrile:H_**2**_O = 1:1) yielded the working solution at concentrations of 0.10, 0.25, 0.50, 0.75, and 1.00 mg/L. Quantification of all compounds was performed with pure commercial standards. Calibration curves were obtained by plotting the peak areas of the compounds to the peak area of the compounds in the standard solution. Calibration curves revealed good linearity, with *R*^*2*^ values exceeding 0.99. Phenolic acids were identified by direct comparison with commercial standards and expessed as mg per 100 g. Xcalibur software (version 2.1) was used for instrument control, data acquisition, and data analysis.

### Chemical assays–antioxidant activity

#### DPPH assay

The DPPH free radical scavenging activities of the extracts were analyzed according to the following procedure [27] with slight modifications. DPPH solution (40 μg mL^**-1**^) was prepared in methanol. To each well on a microtiter plate was added 20 μL of sample (in triplicate) of appropriate concentration (1000, 500, 100, 50, and 10 μg mL^**-1**^) and 180 μL of DPPH solution. As a negative control, 20 μL of methanol was used instead of a sample. BHA, BHT, and AA were used as positive controls. The reaction plates were kept in the dark at a room temperature for 30 minutes, after which the absorbance was measured at 517 nm using Multiskan Sky Thermo Scientific Microtiter plate reader, Finland. The inhibition of DPPH radicals in the test sample was calculated using the following formula and expressed as a percentage (%):

%inhibition=[(Ab‐As)/Ab]×100 (1)

where *Ab* is the absorbance of negative control (without sample) and *As* is the absorbance of the sample at different concentration and the positive controls, as well. The results are presented as the mean of percentage of DPPH radicals inhibition ± standard error.

#### Determination of total reducing potential

The reducing potential of extracts was analyzed by the method [28] and following the procedure [29] with modifications. The reduction capacity is based on the transformation of Fe^**3+**^ to Fe^**2+**^ ferric ions, which results in the development of a blue-green color which intensity can be measured spectrophotometrically. Sample (20 μL, concentrations of 1000, 500, 100, 50, and 10 μg mL^**-1**^), 40 μL of phosphate buffer (0.2 mol L^**-1**^, pH 6.6) and 40 μL of 1% potassium ferricyanide were added to the reaction mixture. The blank was prepared in the same manner as the reaction mixture, with 20 μL of appropriate solvent instead of sample. After an incubation of 20 minutes at 42°C, 40 μL of 10% trichloroacetic acid, 40 μL of distilled water and 8 μL of 0.1% iron trichloride were added. The absorbance was measured at 700 nm after incubation at room temperature for 10 minutes using Multiskan Sky Thermo Scientific, Finland microtiter plate reader. BHA, BHT, and ascorbic acid were used as positive controls and data are presented only against ascorbic acid as the same results were obtained. The reducing potential of the samples is expressed as μmol of ascorbic acid equivalents per gram of dry extract (μmol AAE per g of dry extract).

#### β-carotene/linoleic acid assay

The β-carotene/linoleic acid assay was performed according to a described procedure [30] with modifications. In solution of β-carotene in chloroform (125 μL, 4 mg mL^**-1**^), linoleic acid (6.25 μL), and Tween 40 (50 mg) were dissolved. In the prepared emulsion, 125 μL of chloroform was added. After dissolution, the chloroform was evaporated using a rotary evaporator at 40°C (Buchirotavapor R-114) and the dry residue was dissolved with 25 mL of distilled water. Extracts (concentrations of 1000, 500, 100, 50, and 10 μg mL^**-1**^) and standards (BHA, BHT, and AA) were prepared in triplicate. 200 μL of emulsion and 28 μL of test substance (extracts, standards, 100% methanol–as negative control) were added to each well on the microtiter plate. Absorbance was measured at 490 nm immediately after sample and reagent addition (t_**0**_ = 0) and two hours (t_**120**_ = 120) after incubation using Multiskan Sky Thermo Scientific Microtiter plate reader, Finland. The antioxidant activity of the samples was determined by monitoring the inhibition of β-carotene dye loss, using the following equation:

$$\%\mathrm{inhibition}=\left[\left(A_{120}‐C_{120}\right)/\left(C_{0}‐C_{120}\right)\right]\times 100$$ (2)

Where*A*_*120*_ and *C*_*120*_ were the absorbances measured at t_**120**_ minutes for samples and negative control, respectively, while *C*_*0*_ represent the absorbance of the negative control at t_**0**_. The values obtained are presented as the mean of three replicates ± standard error.

### Biochemical assays–antidiabetic activity

#### α-amylase inhibition assay

The Caraway-Somogy iodine/potassium iodide method, according to Zengin et al [31] was used with modifications. In the test well (*S*), 25 μL of extract (concentration of 1000, 500, 100, 50, and 10 μg mL^**-1**^), 50 μL of α-amylase solution (0.5 mgmL^**-1**^) in sodium phosphate buffer (0.1 mol L^**-1**^, pH 6.8, with 6 mM NaCl) was added. After 10 minutes at 37°C, 50 μL of 0.2% starch was added and the plate was incubated for another 10 minutes at 37°C. The reaction was terminated by the addition of 25 μL of 1mol L^**-1**^ hydrochloric acid, followed by the addition of 100 μL of Lugol’s solution. Acarbose was used as a standard. The absorbance was measured at 630 nm using Multiskan Sky Thermo Scientific, Finland microtiter plate reader. The percentage of inhibition (% *I*) of α-amylase was calculated using the following equation:

$$\%I=\left[\left(S‐B\right)‐C_{1}\right]/C_{2}\times 100$$ (3)

Where enzyme control (*C*_*1*_) contained buffer instead of sample, substrate control (*C*_*2*_) contained buffer instead of enzyme, while the sample color control (*B*) contained buffer instead of enzyme and starch. All measurements were done in triplicates. The results are presented as mean ± standard error.

#### α-glucosidase inhibition test

The potential of extracts in α-glucosidase inhibition was analyzed according to the procedure [32]. pNPG was used as a substrate. In the test well (*S*), 120 μL of extract (concentration of 1000, 500, 100, 50, and 10 μg mL^**-1**^) and 20 μL of enzyme solution (0.5 U mL^**-1**^) in potassium phosphate buffer (0.1 mol L^**-1**^, pH 6.8) were added. After five minutes at 37°C, 20 μL of 5 mM pNPG was added and the plate was incubated for 20 minutes at 37°C. The reaction was stopped by adding 80 μL of 0.2 mol L^**-1**^ sodium carbonate in buffer. Acarbose was used as positive control. The absorbance was measured at 405 nm using Multiskan Sky Thermo Scientific microtiter plate reader, Finland. The α-glucosidase inhibition percentage (% *I*) was calculated using the following equation:

%I=[C‐(S‐B)]/C×100 (4)

where negative control (*C*) contained buffer instead of sample, while sample color control (*B*) contained all components except enzyme, instead of which buffer was added. All measurements were repeated three times. The results are presented as mean ± standard error.

### Biochemical assays–antineurodegenerative activity

#### Acetylcholinesterase inhibition test

The activity of the extracts in acetylcholinesterase inhibition was analyzed based on the procedure [33] with modifications. Briefly, the reaction mixture (*S*) was prepared by adding 140 μL of sodium phosphate buffer (0.1 mol L^**-1**^, pH 7), 20 μL of DTNB solution, 20 μL of extract solution (in buffer containing 5% DMSO, concentrations of 1000, 500, 100, 50, and 10 μg mL^**-1**^) and 20 μL of acetylcholinesterase solution (5 U mL^**-1**^) in Tris-HCl buffer (20 mmol L^**-1**^, pH 7.5). Sample-free mixture was used as a negative control (*C*) and galantamine as a positive control, while the sample color control contained buffer instead of enzyme. Each treatment wasdone in triplicate. The absorbance was measured using Multiskan Sky Thermo Scientific, Finland, microtiter plate reader at a wavelength of 412 nm. The inhibition percentage (*I*) was calculated using the following equation and the results are presented as mean ± standard error:

I(%)=[C−S/C]×100 (5)

#### Tyrosinase inhibition assay

The activity of the samples in tyrosinase inhibition was analyzed according to the slightly modified procedure [34]. The reaction mixture (*S*) was prepared by adding 80 μL of sodium phosphate buffer (0.1 mol L^**-1**^, pH 7), 40 μL of tyrosinase solution (46 U L^**-1**^) and 40 μL of sample (concentrations of 1000, 500, 100, 50, and 10 μg mL^**-1**^). The sample-free mixture was used as a negative control (*C*) and kojic acid as a positive control, while the sample color control contained buffer instead of enzyme. After the addition of 40 μL of L-DOPA buffer solution and incubation for 30 minutes at 25°C, the absorbance was measured using Multiskan Sky Thermo Scientific, Finland, microtiter plate reader at a wavelength of 475 nm. Each treatment was done in triplicate. The inhibition percentage (*I*) was calculated using the following equation and the results are presented as mean ± standard error:

I(%)=[C‐S/C]×100 (6)

### Immunomodulatory effects—antitumor and anti-inflammatory activity

#### Cell line cultivation

To examine the biocompatibility and immunomodulatory effects of moss extracts, four cell lines were cultured: MRC-5 (fibroblast cell line), BV2 (microglia), HCT-116 (colon cancer) and MDA-MB-231 (breast cancer). All cells were purchased from American Tissue Culture Collection (ATCC, Manassas, VA, USA). Cell lines were maintained in culture as monolayers at 37°C in a humidified atmosphere with 5% CO_**2**_, in a complete nutrient medium, with 10% FCS, glucose and 10 μL mL^**-1**^ of antibiotics (penicillin/streptomycin). All cell lines were cultured in culture medium for 24 h and then treated with the corresponding extracts for another 24 h.

#### Cell line treatment

Depending on the number of cells, appropriate dilutions were made for application to a 96-well microtiter plate. Cells were evenly seeded in the appropriate density. The nutrient medium was used as a negative control. The test extracts of *H*. *ciliata* were first dissolved in DMSO, however they were afterwards diluted in DMEM so that their final concentration in the culture was 10 μg mL^**-1**^.

#### MTT assay

The effects of tested extracts on cell viability were determined by MTT assay. To each well 100 μL of cells were added, following 1 × 10^**4**^ cells (MRC-5, BV2, MDA-MB-231) and 5 × 10^**4**^ (HCT-116). For the treatment of MRC-5, HCT-116 and MDA-MB-231 cell lines, 50 μL of the test substance (E1, E2, E3) and 50 μL of full DMEM were added to each well. Control column contained 100 μL of complete DMEM. For the treatment of the BV2 cell line, 50 μL of stimulator—LPS (lipopolysaccharide) and 50 μL of the test substance (E1, E2, E3) were added to each well. Control column contained 50 μL LPS and 50 μL full DMEM. After 24 h incubation, 100 μL of the medium was removed from each well and 10 μL of MTT was added. After 3 h incubation (at 37°C in a humidified atmosphere with 5% CO_**2**_), 100 μL of 10% SDS with 1N hydrochloric acid was added and after the dissolution of the precipitated crystals, the absorbance was measured at 540 nm (Microplate Reader LKB 5060–006, LKB Instruments, Vienna, Austria). The results are presented as a percentage of viability (for MRC-5, HCT-116 and MDA-MB-231) and as metabolic activity (for BV2), from three independent experiments performed in quadruplicate relative to the control (non-treated cell lines) given a value of 100%.

#### NBT assay

In order to determine the effects of tested extracts on the production of ROS and their potential anti-oxidative effects, NBT assay was used. The setup of the experiment was identical to the one used when performing the MTT test. After 24 h incubation, 100 μL of the medium was removed from each well and 10 μL of NBT was added. After 3 h incubation (at 37°C in a humidified atmosphere with 5% CO_**2**_), 100 μL of 10% SDS with 1N hydrochloric acid was added and after the dissolution of the precipitated crystals, the absorbance of the solution was measured at 540 nm (Microplate Reader LKB 5060–006, LKB Instruments, Vienna, Austria). The results are represented as the mean values of the ROS index, calculated based on the absorbance of the samples made in quadruplicate (n = 4) and are expressed as an index relative to the control.

#### Determination of NO production

For the determination of nitric oxide (NO) production, the Griess reaction was applied. Each treatment was done in quadruplicate and there was a control (non-treated cells) for each extract. BV2 cells were stimulated with LPS. 50 μL of cells were seeded and 50 μL of Griess reagent was added to each well (A:B = 1:1). After 10 minutes the absorbance of the solution was measured at 520 nm (Microplate Reader LKB 5060–006, LKB Instruments, Vienna, Austria). After incubation with the test extracts, NO production was calculated (the results are presented as nitrite concentration (μmol L^**-1**^) equivalent to NO production).

### Statistical analysis

The statistical significance of the obtained results was examined using the Statistical Package for Social Sciences program (SPSS) (IBM SPSS Statistics for Windows, Version 25.0., IBM Corporation, Armonk, NY, USA). Within this program, Independent Samples T–Test was used. All numerical parameters are shown as the mean value of the corresponding measurement ± standard error, following: inhibition (%)—anti-enzymatic assays; viability (%) (metabolic activity)—MTT assay; nitrite concentration (μmol L^**-1**^)—Griess assay; Index—NBT assay. The minimum probability value taken as statistically significant is p<0.05.

## Results

### Characterization of selected secondary metabolite content in extracts of *Hedwigia ciliata*

#### Level of total phenolic (acid) contents in extracts

The total phenolic content values of the extracts decrease in the following order: E2>E3>E1. The highest concentration of total phenolics is shown to be in extract E2 and E3, whereas the total phenolic content of the E1 is lower, as shown in Table 2.

**Table 2:** Distribution of secondary metabolites in *Hedwigia ciliata* extracts.

Extract	Solvent	Phenols (mg GAE/g extract)	Phenolic acids (mg CAE/g extract)	Flavonols (mg QE/g extract)	Flavonoids (mg QE/g extract)	Triterpenoids (mg UAE/g extract)
**E1**	Ethanol	11.46 ± 0.48	56.33 ± 5.46	<5	28.75 ± 0.99	134.8 ± 4.29
**E2**	Water:Ethanol	16.04 ± 0.29	57.56 ± 2.84	<5	20.60 ± 0.90	94.38 ± 0.43
**E3**	Ethyl acetate	14.04 ± 0.42	151.14 ± 7.52	15.50 ± 0.99	49.54 ± 3.55	79.37± 2.61

Based on the obtained results, the best extracting solvent was the mixture of water:ethanol (50:50, vol%). According to the obtained results, extracts E1 and E2 exhibited similar phenolic acid content, while the highest concentration of phenolic acids was shown to be in E3 extract, which indicated a significantly higher level of these compounds than in the other tested extracts.

#### Level of total flavonol and flavonoid contents in extracts

The results presented in Table 2 have shown that flavonols were only present in extract E3, while low content was observed in E1 and E2. Experimental data presented in Table 2 also demonstrated that the concentration of total flavonoids varied among different extracts. The values decrease in the following order: E3>E1>E2. The highest concentration of flavonoids was found in extract E3, almost twice the value of the other solvents, E1 and E2.

#### Level of total triterpenoid and coumarin contents in extracts

The highest triterpenoid content was obtained in E1, whereas the lowest total triterpenoid content occurred in E3 (Table 2). According to the obtained results (data not shown), no traces of coumarins were found in the tested extracts.

#### Liquid chromatography–mass spectrometry analyses of extracts

Using the Liquid Chromatography—Mass Spectrometry (LC/MS) analyses, the presence of different phenolic and flavonoid compounds was determined and it was observed that they differed among the corresponding extracts depending on their polarity. Based on the obtained results, extracts E1 and E2 showed higher concentration of phenolic compounds compared to E3 extract. In the ethanolic (E1) extracts, the most prevalent was shown to be protocatechuic acid, while for the E2 extract measured value was half the previous value. The second most common compound was *p*-hydroxybenzoicacid (Table 3). Other compounds such as gallic acid, caffeic acid, quercetin 3-*O*-rutinoside, apigenin, naringenin and kaempferol were also detected in E1 and E2 extract, however at lower concentrations, ranging from 1.00–2.45 g per 100 g. In contrast, the values for the other tested compounds were lower than 1.00 g per 100 g in the less polar, E3 extract.

**Table 3:** LC/MS analyses of the extracts.

[mg per 100 g weight]	E1	E2	E3
Gallic acid	1.48	1.16	0.49
Protocatechuic acid	8.11	4.01	0.83
5-*O*-Caffeoylquinic acid	0.12	0.08	0.02
*p*-Hydroxybenzoic acid	6.97	9.21	0.60
Caffeic acid	1.04	0.51	0.08
Quercetin 3-*O*-rutinoside	0.87	1.29	0.03
*p*-Coumaric acid	0.99	0.42	0.26
Quercetin 3-*O*-glucoside	0.69	0.23	0.14
Isorhamnetin 3-*O*-glucoside	0.37	0.16	0.23
Eriodictyol	0.46	0.38	0.07
Apigenin	1.83	0.86	0.15
Naringenin	2.42	1.09	0.07
Kaempferol	2.45	1.00	0.10
Acacetin	0.23	0.08	0.04

### Antioxidant potential of the *Hedwigia ciliata* extracts

Antioxidant activity of moss extracts was analyzed using DPPH assay. The data obtained exhibited low to moderate free radical scavenging activity. Antioxidant activity was only detected at the concentration of 1000 μg mL^**-1**^ (S1 Table). The highest DPPH radical scavenging activity was measured for the extract E2. Regarding extracts E1 and E3, low inhibitory effects were measured, with no significant difference between them. However, considering positive controls, this method did not display any significant capacity of the extracts in quenching DPPH radicals.

The total reducing potential assay did not show any antioxidant effect of the tested extracts. None of the samples had a significant influence on the transformation of Fe^**3+**^ → Fe^**2+**^ ferric ions. The results from this study showed that no relevant reducing capacity of the selected extracts was detected (S2 Table).

**Fig 2:**
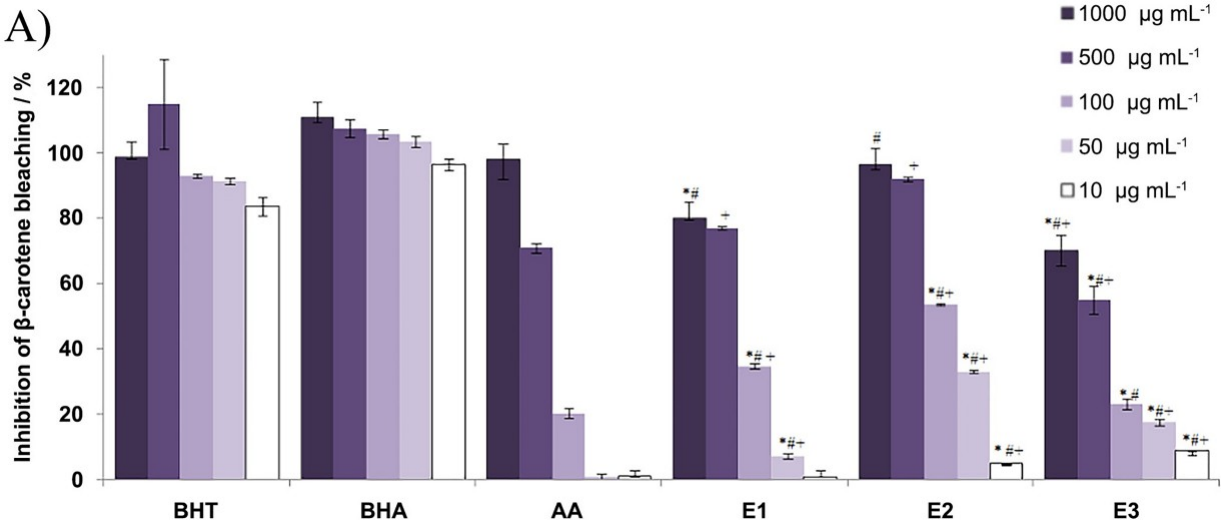
Antioxidant activity–the capacity of the extracts (E1, E2, E3) to inhibit lipid peroxidation by measuring the inhibition rate of ß-carotene bleaching (%). The results are expressed as the inhibition percentage, as mean value ± SE from an experiment performed in triplicate: * p<0.05; different concentrations of extracts *vs*. BHT (3,5-di-tert-butyl-4-hydroxytoluene); # p<0.05; different concentrations of extracts *vs*. BHA (2-tert-butyl-4-hydroxyanisole); + p<0.05; different concentrations of extracts *vs*. AA (ascorbic acid).

On the other hand, using β-carotene/linoleic acid assay, significant antioxidant properties of all the extracts were exhibited. This method is based on the discoloration of the yellowish color of β-carotene solution due to the breaking of π-conjugation by lipid or lipid peroxyl radicals formed by linoleic acid autoxidation [35]. The β-carotene/linoleic acid assay determines the capacity of the extracts to inhibit lipid peroxidation by measuring the inhibition of β-carotene bleaching. As shown in Fig 2, all the extracts displayed high activity in a concentration-dependent manner. They significantly inhibited the bleaching of β-carotene in comparison with positive controls (BHT, BHA, and AA). At the concentrations of 100, 50, and 10 μg mL^**-1**^, extracts performed better inhibition rate than ascorbic acid. The highest activities were found for E2 extract, but also significant inhibition was measured for E1 and E3 extracts. The antioxidant activity of the extracts decreased in the following manner: E2>E1>E3.

### Antidiabetic activity of the *Hedwigia ciliata* extracts

**Fig 3:**
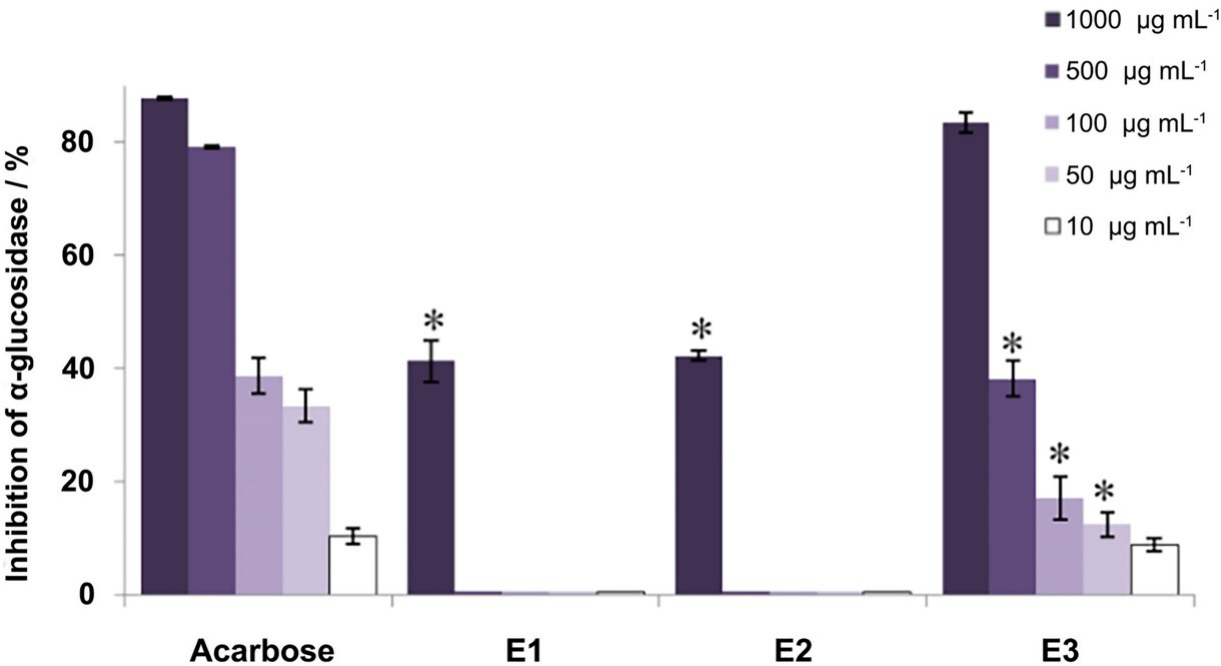
Antidiabetic activity–effects of the extracts (E1, E2 and E3) against α-glucosidase. The results are expressed as the inhibition percentage, as mean value ± SE from an experiment performed in triplicate (* p<0.05; different concentrations of extracts *vs*. corresponding standard, acarbose).

The inhibitory effects of the extracts against α-amylase and α-glucosidase activities were investigated as antidiabetic potential of these moss extracts. The results revealed that none of the extracts showed effects against the α-amylase activity, the inhibition percentage was less than 5% (S3 Table). In contrast, all of the tested extracts exhibited inhibitory effect against α-glucosidase (Fig 3). Among the analyzed extracts, the highest inhibition of the α-glucosidase activity was detected for E3 extract. All of the tested concentrations (1000, 500, 100, 50, and 10 μg mL^**-1**^) exerted remarkable inhibitory effects in a concentration dependent manner with statistically significant values (p<0.05) obtained for the concentrations of 500, 100, and 50 μg mL^**-1**^. On the other hand, E1 and E2 extracts at the concentration of 1000 μg mL^**-1**^ exhibited lower potential for inhibition of α-glucosidase activity.

### Antineurodegenerative and anti-neuroinflammatory activity of the *Hedwigia ciliata* extracts

#### Antineurodegenerative activity

**Fig 4:**
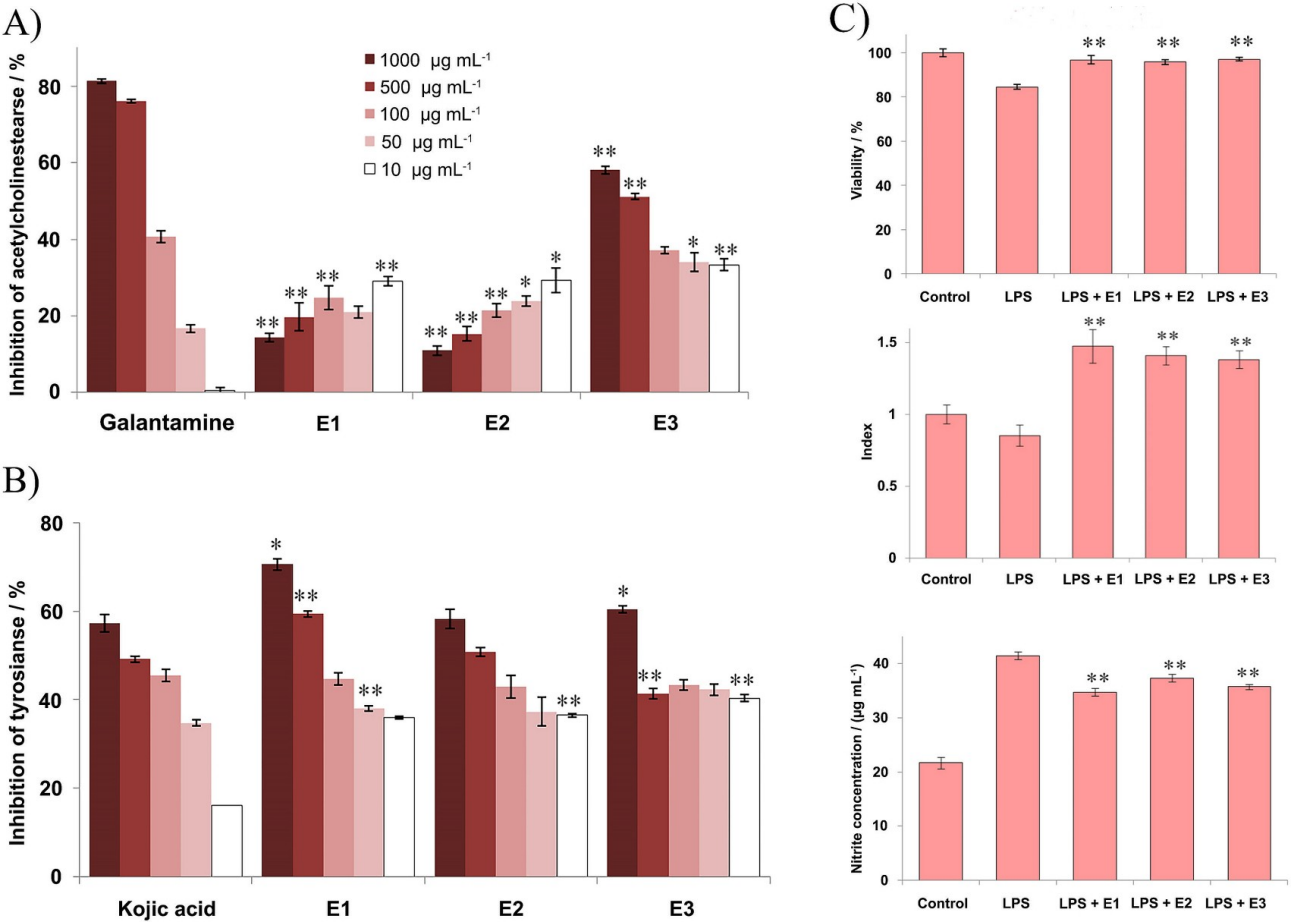
Antineurodegenerative and anti-neuroinflammatory activity–effects of the corresponding extracts (E1, E2, E3) towards acetylcholinesterase (A) and tyrosinase (B). The results are expressed as the inhibition percentage, as mean value ± SE from an experiment performed in triplicate (* p<0.05; ** p<0.01 different concentrations of extracts *vs*. corresponding standard, galantamine or kojic acid). (C) Anti-neuroinflammatory activity of the extracts towards BV2 cell line–the effect of investigated extracts on the viability, NO production (measured by nitrite level), and ROS production *in vitro* after 24 h of exposure. The results are expressed as the mean ± SE from a representative experiment of three independent experiments performed in quadruplicate (** p<0.01 extract *vs*. LPS-treated cells).

The results obtained regarding the acetylcholinesterase inhibitory activity are given in the Fig 4A. The data are similar for the all tested extracts, showing moderate to low inhibitory effect in comparison with the positive control, galantamine. The highest acetylcholinesterase inhibition among analyzed samples was noted for the E3 extract. As presented in Fig 4A, all of the tested concentrations of this extract exerted moderate inhibitory activity, in a concentration-dependent manner. Regarding E1 and E2 extracts, notable inhibitory effects were also measured. Interestingly, the obtained inhibition values increased proportionally with the decreasing of sample concentration (Fig 4A). As presented in Fig 4B, all of the extracts exhibited notable effects against tyrosinase, with higher inhibition rate in comparison with the positive control, kojic acid. The most potent inhibitory effects were observed for E1 extract, with a maximum of inhibition at a concentration of 1000 μg mL^**-1**^, which was higher than inhibition value of the kojic acid. The extracts E2 and E3 also showed noticeable inhibitory effects for the highest sample concentration used (Fig 4B), although statistically insignificant for the E2 extract. The values obtained for the other concentrations displayed significant inhibition, and they are decreasing proportionally with the decrease in sample concentration.

#### Anti-neuroinflammatory activity

Activated microglia release mediators such as inflammatory molecules or neurotrophic factors that display either harmful or beneficial effects on neuronal signaling and survival [36]. The amounts of released factors depend on the intensity of stimulation and on time; therefore we first determined whether microglial cells modulate the metabolic activity and the production of ROS and NO, by following activation by LPS, in a concentration- or a time-dependent manner. For this purpose, microglial cells BV2 were stimulated for 24 h with LPS. Microglial activation was confirmed by an increase in nitrite concentration in the cell culture supernatants (Fig 4C). Regarding the treatment of BV2 cells, as presented in the Fig 4C, the metabolic activity/viability of LPS-stimulated BV2 cells was slowly reduced in comparison with the control non-stimulated cells. After simultaneous treatment with LPS and extracts, a significant normalization in the metabolic activity of BV2 cells was detected, with corresponding extracts showing similar results.

As expected, LPS-stimulated BV2 cells produced large amounts of NO (Fig 4C) compared with the non-treated cells. The results have shown that all of the extracts expressed similar effects in reducing NO production by LPS-stimulated BV2 cells, indicating their potential anti-neuroinflammatory activity.

Furthermore, the ROS production also increased by LPS-stimulated BV2 cells after treatment with the tested extracts in selected concentration (10 mg mL^**-1**^) as presented in Fig 4C. All of the analyzed extracts performed similar effects, suggesting a potential of the selected extracts for increasing of antioxidant mechanisms in investigated cells including ROS production.

### Antitumor activity of the *Hedwigia ciliata* extracts

The obtained results (S4 Table) have shown that all the examined extracts do not inhibit the proliferation of these cells. None of the extracts exhibited any cytotoxic effects towards MRC-5 cells, since the viability of the treated cells was higher than 90%. The extracts were found to be biocompatible, as they did not shown inhibition of the viability and proliferation of normal human cells.

Using the MTT assay, the effects of moss extracts of *H*. *ciliata* on the viability of two tumor cell lines (MDA-MB-231 and HCT-116) were also examined.

**Fig 5:**
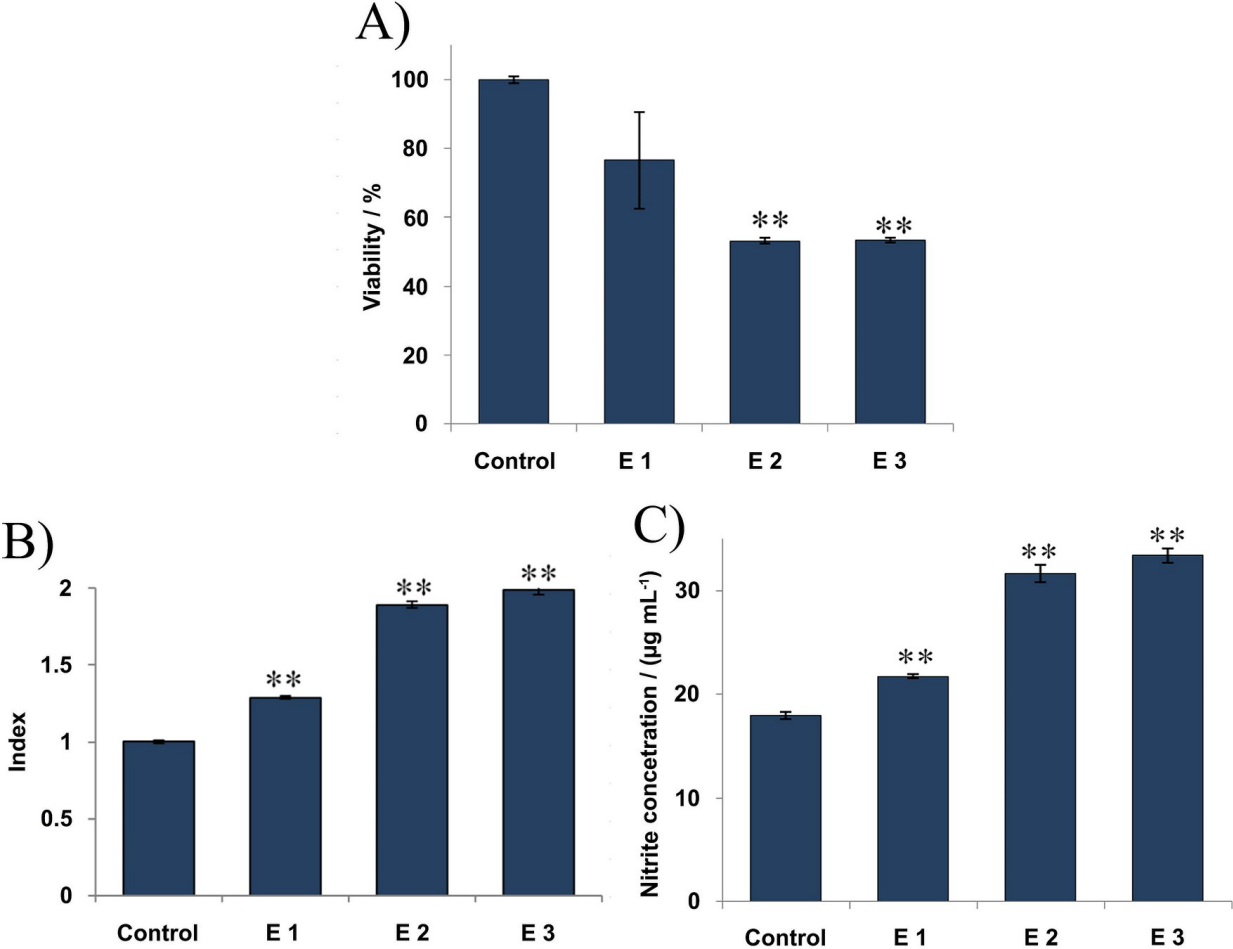
Antitumor activity of the extracts towards MDA-MB-231 cell line *in vitro* after 24 h of exposure; (A) MTT test–The effect of investigated extracts on the viability; (B) NBT test–The effect of investigated extracts on ROS production; (C) NO test–The effect of investigated extracts on NO production (measured by nitrite level). The results are expressed as the mean ± SE from a representative experiment of three independent experiments performed in quadruplicate (*p<0.05; ** p<0.01 extract *vs*. non-treated control cells).

The data showed that the tested extracts were not effective in inhibiting HCT-116 cell proliferation; no antiproliferative effect was detected, as the cell viability (%) did not change after treating with test extracts (S5 Table). On the other hand, a significant inhibition of proliferation against the MDA-MB-231 cell line (Fig 5A) was observed. All three extracts showed antiproliferative/antitumor activity against MDA-MB-231 cells, among which E2 and E3 extracts exhibited the highest activity (higher than 50% of inhibition). Nevertheless, slightly significant reduction of the MDA-MB-231 cell viability was measured for the extract E1.

Using the NBT test, the effect of test extracts on ROS production was examined. The obtained results revealed that none of the tested extracts showed a significant change in ROS generation by HCT-116 cells, while modulation in ROS production by MDA-MB-231 cells has been detected. The data presented in Fig 5B showed that the extracts increased the ROS production by MDA-MB-231 cells in comparison with the control cells with index 1. All of the analyzed extracts performed similar effects, suggesting a potential of the selected extracts for increasing of antioxidant mechanisms in MDA-MB-231 cells including ROS production.

Furthermore, extracts were evaluated for their NO production activity against HCT-116 and MDA-MB-231 cells using the Griess assay. Regarding MDA-MB-231 cell line, the obtained results showed increased NO production by these cells (Fig 5C) in comparison with the non-treated cells.

## Discussion

Phenolic compounds, together with terpenoids represent one of the major plant constituents which compose a very broad and heterogeneous class of bioactive molecules, with large structural diversity and are considered to have antioxidant, antiproliferative, antimicrobial, and anti-inflammatory effect [4]. Studies have shown the presence of different phenolic compounds, as well as terpenoids, that are widely distributed in mosses [37]. The chemical characterization of *H*. *ciliata* extracts included the determination of total content of phenols, phenolic acids, flavonoids, flavonols, triterpenoids, and coumarins. In analyzing the bioactivity of compounds, it is also very important to consider factors such as the extraction method and the choice of the appropriate solvent, as well as the variation of the active components of tested plants depending on the season and locality of sampling. Based on their solubility in various solvents, the extracts showed a different secondary metabolite content. The concentration of total phenols was similar in all of the three solvents used, where the highest observed concentration was for water:ethanol (50:50, vol%) extract. Due to the presence of the hydroxyl group (-OH) in these compounds, water and ethanol can build hydrogen bonds with these compounds and easily dissolve them. On the contrary, the concentration of phenolic acids, flavonoids, and flavonols was the highest in the less polar, ethyl acetate extract.

Triterpenoids represent a wide group of natural compounds with great structural diversity and are often present in a glycosylated form (saponins) in plants [38]. As the highest triterpenoid content was shown for 96% ethanol and water:ethanol (50:50, vol%) extracts, it may imply that glycosylated triterpenes are present in the examined moss, as in literature they are generally characterized as water soluble [38]. Many studies have shown that terpenoids are potential bioactive molecules that can be used in cancer therapy, as they can inhibit cancer cell proliferation, with triterpenoids being the major class with antitumor potential [39]. Studies have shown that triterpenoids are particularly potent against breast cancer cell lines [40,41] which may indicate they are involved in antiproliferative effects towards MDA-MB-231 cells in the present study.

LC/MS analyses indicated that 96% ethanol and water:ethanol (50:50, vol%) extracts of *H*. *ciliata* contain a diverse group of phenolic and flavonoid compounds, with the highest concentrations measured for protocatechuic and p-hydroxybenzoic acids. The significance of the two acids have been described in many papers, which highlighted their antitumor activity and potential induction of apoptosis in various tumor cell lines, including the breast cancer tumor cell line [42,43] which was examined in this study and achieved 50% of inhibition rate of MDA-231-MB cells. Furthermore, many studies revealed anti-inflammatory, antioxidant, as well as the neuroprotective effect of these two phenolic acids [44–46] which may suggest that they are involved in the biological activity of the corresponding moss extracts as these immunomodulatory effects were also revealed in the present study.

Natural compounds are known as powerful antioxidants which can aid in preventing or delaying the oxidative stress and the damage of biomolecules such as DNA, proteins or lipids, caused by ROS [47]. The antioxidant potential of examined compounds is related to their chemical structure which can range from simple monomers to highly polymerized structures that can occur as aglycones or glycosides [48]. The position and number of functional groups, the arrangement of aromatic rings, hydrogen bonds, conjugation and resonance, directly impact their antioxidant activity [49]. The most important structural feature that is responsible for the antioxidant properties is the hydroxyl group. Hydroxyl groups represent good hydrogen donors which can reduce or inhibit free radicals, while the electrons of benzene rings contribute to the stabilization of produced radicals [50]. The free radical scavenging and antioxidant activities are based on different reaction mechanisms that may include H-atom transfer or electron transfer, depending on phenolics structure [51]. The antioxidant potential is also affected by the polarity of the used solvent, as it can interact with the hydroxyl groups and consequently forms additional hydrogen bonds.

By using three different methods (DPPH, total reducing potential and β-carotene/linoleic acid assay), the antioxidant activity of the extracts was tested, however significant activity was only measured for β-carotene/linoleic acid assay, where the highest activity was found for water:ethanol extracts. Different methods may show distinctive results as these assays are based on diverse reaction mechanisms such as the ability of an antioxidant to donate an electron, free radical scavenging or inhibition of lipid hydroperoxides [47]. Many studies reported the “polar paradox” which is a phenomenon that describes oddly behaviour of the antioxidants in different media. It was shown that polar antioxidants are more effective in less polar media, while non-polar, lipophilic antioxidants are more potent in relatively more polar media, such as oil-in-water emulsions [52]. These features should be considered when comparing different methods for antioxidant activity evaluation. The results presented in this study suggest that the antioxidant activity of the extracts may depend on the polarity of the media. Moreover, extracts are usually complex mixtures consisting of a variety of compounds which differ in chemical structures, positions of functional groups and often shows synergic effects and mutual interaction [50]. Nonetheless, *in vitro* assays for determining the antioxidant activity may differ from *in vivo* tests, as antioxidant capacity also depends on the lifespan of active compounds, which must be taken into account when considering the overall antioxidant potential of a substance [47]. Therefore, the antioxidant effect of *H*. *ciliata* extracts in *in vivo* animal models remains to be explored. It is possible because the biocompatibility testing of these extracts demonstrate high biocompatibility and making these extracts promising candidates for natural supplement or basic for some new drug development.

All of the tested extracts exhibited inhibitory effect against α-glucosidase with the highest inhibition detected in ethyl acetate extract, while none of the extracts showed effects against the α-amylase activity. Chemical structures of the active compounds that are present in extracts, as well as the presence of different amino acid residues that are involved in the catalytic reactionbyα-glucosidase and α-amylase, greatly impact the inhibitory potential of the extracts [53]. The potential mechanism of action could be through the formation of hydrogen bonds between hydroxyl groups of the inhibitory compounds and catalytic residues in the active site of the α-glucosidase and α-amylase [53,54]. Binding to the active site induces conformational changes of enzymes, causing loss of function. Future studies are welcome to evaluate the conformational changes of α-glucosidase after treatment with ethyl acetate extract of *H*. *ciliata* as well as beneficial supplement in patients with diabetes mellitus.

By analyzing the inhibition of acetylcholinesterase and tyrosinase, the neuroprotective potential of the extracts was evaluated. The inhibition of acetylcholinesterase enzyme is important for the treatment of Alzheimer’s disease, while tyrosinase inhibition is effective in Parkinson disease treatment. Alzheimer’s disease is distinguished by deficiency of a neurotransmitter acetylcholine. Acetylcholinesterase inhibitors prevent breaking down of acetylcholine and consequently increasing the level of acetylcholine, as well as the duration of the neurotransmitter action [55]. Tyrosinase catalyzes the hydroxylation of tyrosine (monophenol) to 3,4-dihydroxyphenylalanine or DOPA (o-diphenol) and the conversion of DOPA to the corresponding o-quinone [56]. The mechanism of action by which inhibitors bind to the enzyme may involve chelation of the copper ions that are present in the active site, and therefore hinder the tyrosinase activity [57]. Regarding the acetylcholinesterase inhibitory activity, the highest inhibition among analyzed samples was evaluated for the ethyl acetate extract which may suggest that bioactive compounds are of more lipophilic nature. That is consistent with the fact that small lipofilic molecules can easily cross the blood-brain barrier by transmembrane diffusion, and consequently inhibit acetylcholinesterase activity [58]. Interestingly, the obtained inhibition values were increasing proportionally with the decrease in sample concentration, which can be caused by the mutual interaction of different compounds at higher concentrations. All of the tested extracts exhibited inhibitory effects against tyrosinase, however the most potent was the ethanolic extract. As the protocatechuic acid was the most prevalent in this extract, inhibitory effects may be attributed to this compound, as previous papers have also reported its inhibitory activity towards tyrosinase [59,60].

Recent studies have shown that phenolics, flavonoids, flavonols, together with terpenoids, are effective in inhibition of acetylcholinesterase, tyrosinase,α-glucosidase, and α-amylase [61–66]. Because of the variety of compounds and their chemical structures, a different type of interactions between inhibitors and the amino acid residues of the enzyme active site can occur, including hydrogen bonds, van der Waals forces, and hydrophobic interactions. The synergistic effects of these compounds may contribute to the overall antidiabetic, along with neuroprotective properties. As ethyl acetate extract demonstrated the best inhibition rate for α-glucosidase and acetylcholinesterase, the active components may be of less polar nature. Further studies are necessary in order to identify the active compounds and determine the type of inhibition, which will aid in the elucidation of the mechanism of action and design of potential therapeutics.

In context with neurodegeneration and development of neuroprotective molecules it is important to emphasize that microglia play an substantial role in neuroinflammation. Activated microglia secrete large amounts of different pro-inflammatory molecules including NO [67]. The inflammatory responses are regulated through various regulation mechanisms and signaling pathways including the NF-κB signaling pathway, which plays a major role in controlling the production of iNOS, enzyme which subsequently induces the production of NO. Excess NO can generate apoptosis of macrophages, but also can cause neurodegenerative diseases, such as and Parkinson disease [68,69]. Several studies indicated potential use of polyphenolic compounds for the treatment of neuroinflammation, by regulation of a TLR4 (receptor for LPS) that is in bases of triggering that process. This way, bioactive compounds may interfere with various signaling pathways including TLR4/NF-κB/STAT, TLR4-MAPK/NF-κB, and TLR4/MyD88/NF-κB signaling cascade. Via these signaling pathways, extracts may exert neuroprotective effects, inhibit the production of inflammatory mediators, and reduce neuronal apoptosis mediated by activated microglia [70,71]. Future studies are necessary in order to identify the signaling pathway affected by these extracts. In agreement with the hypothesis that *H*. *ciliata* extracts affect (inhibit) TLR4/NF-κB signaling mechanism is the capacity of extracts to down-regulate NO production which is the result of NF-κB signaling activation. On the other hand, absence of extracts activity towards ROS production by LPS-stimulated microglia is in line with the results observed for the antioxidant potential of the extracts. As shown previously by DPPH and total reducing potential assays, extracts seem to have a low capacity of free radical scavenging, which may be the cause of their inability to decrease the concentration of ROS as pro-inflammatory molecules. As neuroinflammation and increased levels of ROS characterizes neurodegenerative disorders [72] precise mechanisms of these extracts that may promote such response remain unclear.

This study showed for the first time that extracts of moss *H*. *ciliata* exhibited antitumor activity against the MDA-MB-231cells. As no antitumor effect was detected against HCT-116 cells, it can be concluded that there is a difference in the proliferation preference of certain tumor cells, which needs to be further investigated. Tumor cells of different origins are characterized by differentially regulated molecular mechanisms and signaling pathways, which may account for the different effect of the extract towards the MDA-MB-231 and HCT-116 cell lines. Earlier studies indicated the ability of phenolic and other compounds to prevent and/or slow the progression of tumor cells *in vitro* [73,74]. The activity of these compounds is reflected in their interaction with the basic cellular processes related to cell proliferation, differentiation, inflammation, apoptosis, angiogenesis, etc. The mechanism of action of these processes is based on the regulation of intracellular signal transduction and gene expression [75].

Numerous studies have reported antiproliferative properties of the phenol and flavonoid compounds, together with terpenoids, which are one of the major secondary metabolites present in bryophytes [73]. Phenolic compounds, including 4-hydroxybenzoic acid, which was observed in high concentrations for water:ethanol extract, has been demonstrated to induce apoptosis in breast cancer cell lines [43]. In addition, protocatechuic acid also has been reported to have anti-metastatic potential [42]. Among a very diverse group of potentially active compounds, flavonoids have been identified as one of the most potent antitumor agents [76]. Recent research suggests that flavonoids may affect the regulation of the cell cycle, fatty acid synthesis, and apoptosis [77]. The antiproliferative activity has been attributed to the flavonoids such as apigenin, kaempferol, and quarcetin [77] which were also detected in the extracts of *H*. *ciliata* investigated in this study. Different molecular mechanisms of action have been described, especially the ability of flavonoids to inhibit the NF-κB signaling pathway and NF-κB regulated gene expression, which may enhance the induction of apoptosis [78]. Also, a great number of terpenoid compounds has been described as effective inhibitors of tumor growth, preeminently towards human breast cancer cells [40,41] that is in line with results presented in this study. It was shown that these compounds can regulate several molecular processes that are involved in the apoptosis induction, including inhibition of the ubiquitin-proteasome, NF-κB pathways, antiapoptotic protein Bcl-2 and the heat shock proteins [41]. Also, triterpenoids which we examined in this study, are close to steroids in structure and has been linked to hormone-related cancers [39] such as may be breast cancer. As ethyl acetate extract exhibited the highest rate of inhibition of MDA-MB-231 cells, antitumor activity may be attributed to compounds that are of lipophilic nature. However, further research is necessary to identify active compounds and the mechanisms underlying antitumor processes, such as the affect to NF-κB signaling the main mechanism of action of these extracts for noted anti-neuroinflammatory and antiproliferative effects.

Antiproliferative activity of the extracts may correlate with these results as various studies reported the ROS and NO-mediated antitumor activity [79–81]. One of the mechanisms included in the inhibition of tumor cells growth is the induction of apoptotic pathways. Several papers showed that the apoptosis in MDA-MB-231 cells is promoted by ROS generation [82]. Pro-apoptotic effects of NO has also been demonstrated and are associated with cytochrome C release and increase in the p53 expression [83]. Although further studies on the mechanism of apoptotic pathways are needed, these results suggest that antiproliferative effects of these extracts toward MDA-MB-231 cells may be caused by ROS and NO increased production.

## Conclusions

This study provides a new insight into the chemical and biological characterization of extracts of the moss *H*. *ciliata* from the Vršačke Planine Mts., Nature park, Serbia. The chemical characterization of the extracts revealed a wide range of phenolic, flavonoid, and triterpenoid compounds as secondary metabolites of high biological activity. Furthermore, the extracts demonstrated the antioxidant activity via β-carotene/linoleic acid assay, as well as an inhibitory effect against α-glucosidase, acetylcholinesterase, and tyrosinase. In this study, it is reported for the first time that the *H*. *ciliata* extracts exerted an anti-neuroinflammatory activity toward LPS-activated BV2 cells by decreasing NO production, along with the significant antiproliferative activity against MDA-MB-231 cells (~ 50% inhibition). The literature data together with this research indicate the presence of bioactive potential of *H*. *ciliata* extracts, including antioxidant, antidiabetic, antineurodegenartive/anti-neuroinflammatory (neuroprotective), and antitumor activities. However, further studies are needed to investigate the additional and detailed mechanisms of action underlying immunomodulatory processes, as well as evaluation of extracts activity *in vivo*. Most importantly, different moss extracts could provide entirely new avenues for developing more efficient natural supplements for preventing and treating different inflammatory/degenerative and malignant processes.

## Supporting information

S1 Table The DPPH (2, 2-diphenyl-1-picrylhydrazyl) free radical scavenging activities of the corresponding extracts E1 (96% ethanol), E2 (water:ethanol– 50:50, vol%) and E3 (ethyl acetate) in comparison with the standards BHT (3, 5-di-tert-butyl-4-hydroxytoluene), BHA (2-tert-butyl-4-hydroxyanisole) and AA (ascorbic acid). The results are represented as inhibition (%) of DPPH radical scavenging. The results are expressed as the mean ± SE relative to a non-treated control cells from an experiment performed in triplicate.
(DOCX)

S2 Table Total reducing power (TRP) of the corresponding extracts E1 (96% ethanol), E2 (water:ethanol– 50:50, vol%), and E3 (ethyl acetate) in comparison with the standards BHT (3,5-di-tert-butyl-4-hydroxytoluene), BHA (2-tert-butyl-4-hydroxyanisole) and AA (ascorbic acid). The reduction potential of the samples is expressed as μmol of ascorbic acid (C6H8O6) equivalents per gram of dry extract (μmol AAE/g of dry extract). The results are expressed as the mean ± SE relative to a non-treated control cells from an experiment performed in triplicate.
(DOCX)

S3 Table The effects of the corresponding extracts E1 (96% ethanol), E2 (water:ethanol– 50:50, vol%), and E3 (ethyl acetate) against α-amylase, in comparison with the standard–acarbose. The results are presented as percentage of inhibition (%) of α-amylase. The results are expressed as the mean ± SE relative to a non-treated control cells from an experiment performed in triplicate.
(DOCX)

S4 Table The antiproliferative and anti-inflammatory effects of the corresponding extracts (10 μg/mL), E1 (96% ethanol), E2 (water:ethanol– 50:50, vol%), and E3 (ethyl acetate) on the MRC-5 cell line determined by MTT, NBT, and Griess test. The results are expressed as the mean ± SE relative to a non-treated control cells from a representative experiment of three independent experiments performed in quadruplicate.
(DOCX)

S5 Table The antiproliferative and anti-inflammatory effects of the corresponding extracts (10 μg/mL), E1 (96% ethanol), E2 (water:ethanol– 50:50, vol%), and E3 (ethyl acetate) on the HCT-116 cell line determined by MTT, NBT and Griess test. The results are expressed as the mean ± SE relative to a non-treated control cells from a representative experiment of three independent experiments performed in quadruplicate.
(DOCX)
