# Supplementary material for: Chemical characterization and in vitro immunomodulatory effects of different extracts of moss Hedwigia ciliata (Hedw.) P. Beauv. from the Vršačke Planine Mts., Serbia
Source: PLoS One. 2021 Feb 11;16(2):e0246810. doi: 10.1371/journal.pone.0246810 (PMC7877662; doi:10.1371/journal.pone.0246810)
Supplement: S1 Table — The results are represented as inhibition (%) of DPPH radical scavenging. The results are expressed as the mean ± SE relative to a non-treated control cells from an experiment performed in triplicate. (DOCX) [file pone.0246810.s001.docx]

**S1 Table.** The DPPH(2,2-diphenyl-1-picrylhydrazyl)free radical scavenging activities of the corresponding extracts E1 (96 % ethanol), E2 (water:ethanol – 50:50, vol%) and E3 (ethyl acetate) in comparison with the standards BHT (3,5-di-tert-butyl-4-hydroxytoluene), BHA (2-tert-butyl-4-hydroxyanisole) and AA (ascorbic acid). The results are represented as inhibition (%) of DPPH radical scavenging.The results are expressed as the mean ± SE relative to a non-treated control cells from an experimentperformed in triplicate.

| **Concentration (μgmL^-1^)** |  |  | **Inhibition (%)** |  |  |  |
| --- | --- | --- | --- | --- | --- | --- |
|  | **E1** | **E2** | **E3** | **BHT** | **BHA** | **AA** |
| 1000 | 12 ± 0.2 | 23 ± 0.6 | 8 ± 0.4 | 84 ± 0.2 | 85 ± 0.6 | < 5 |
| 500 | < 5 | < 5 | < 5 | 72 ± 0.3 | 80 ± 0.7 | < 5 |
| 100 | < 5 | < 5 | < 5 | 26 ± 0.9 | 30 ± 1.3 | < 5 |
| 50 | < 5 | < 5 | < 5 | 13 ± 0.7 | 15 ± 0.5 | < 5 |
| 10 | < 5 | < 5 | < 5 | < 5 | < 5 | 8 ± 0.1 |
