# Supplementary material for: Chemical characterization and in vitro immunomodulatory effects of different extracts of moss Hedwigia ciliata (Hedw.) P. Beauv. from the Vršačke Planine Mts., Serbia
Source: PLoS One. 2021 Feb 11;16(2):e0246810. doi: 10.1371/journal.pone.0246810 (PMC7877662; doi:10.1371/journal.pone.0246810)
Supplement: S2 Table — The reduction potential of the samples is expressed as μmol of ascorbic acid (C6H8O6) equivalents per gram of dry extract (μmol AAE/g of dry extract). The results are expressed as the mean ± SE relative to a non-treated control cells from an experiment performed in triplicate. (DOCX) [file pone.0246810.s002.docx]

**S2 Table.** Total reducing power (TRP) of the corresponding extracts E1 (96 % ethanol), E2 (water:ethanol – 50:50, vol%), and E3 (ethyl acetate) in comparison with the standards BHT (3,5-di-tert-butyl-4-hydroxytoluene), BHA (2-tert-butyl-4-hydroxyanisole) and AA (ascorbic acid). The reduction potential of the samples is expressed as μmol of ascorbic acid (C_6_H_8_O_6_) equivalents per gram of dry extract (μmol AAE/g of dry extract).The results are expressed as the mean ± SE relative to a non-treated control cells from an experiment performed in triplicate.

| **Concentration**  **(μgmL^-1^)** |  |  | **TRP** |  |  |  |
| --- | --- | --- | --- | --- | --- | --- |
|  | **E1** | **E2** | **E3** | **BHT** | **BHA** | **AA** |
| 1000 | 8 ± 0.1 | 10 ± 1.5 | < 5 | 139 ± 1.5 | 119 ± 9.6 | 130 ± 1.5 |
| 500 | < 5 | < 5 | < 5 | 123 ± 1.0 | 121 ± 10.1 | 129 ± 3.0 |
| 100 | < 5 | < 5 | < 5 | 58 ± 1.6 | 50 ± 2.5 | 81 ± 2.9 |
| 50 | < 5 | < 5 | < 5 | 30 ± 1.2 | 27 ± 2.0 | 59 ± 2.3 |
| 10 | < 5 | < 5 | < 5 | 10 ± 1.5 | 5 ± 0.7 | < 5 |
