# Supplementary material for: Chemical characterization and in vitro immunomodulatory effects of different extracts of moss Hedwigia ciliata (Hedw.) P. Beauv. from the Vršačke Planine Mts., Serbia
Source: PLoS One. 2021 Feb 11;16(2):e0246810. doi: 10.1371/journal.pone.0246810 (PMC7877662; doi:10.1371/journal.pone.0246810)
Supplement: S3 Table — The results are presented as percentage of inhibition (%) of α-amylase. The results are expressed as the mean ± SE relative to a non-treated control cells from an experiment performed in triplicate. (DOCX) [file pone.0246810.s003.docx]

**S3 Table.** Theeffects of the corresponding extracts E1 (96 % ethanol), E2 (water:ethanol – 50:50, vol%), and E3 (ethyl acetate) against α-amylase, in comparison with the standard – acarbose.The results are presented as percentage of inhibition (%) of α-amylase. The results are expressed as the mean ± SE relative to a non-treated control cells from an experiment performed in triplicate.

| **Concentration (μgmL^-1^)** |  |  | **Inhibition (%)** |  |
| --- | --- | --- | --- | --- |
|  | **E1** | **E2** | **E3** | **Acarbose** |
| 1000 | < 5 | < 5 | 5.3 ± 0.1 | 83.0 ± 1.2 |
| 500 | < 5 | < 5 | < 5 | 80.2 ± 1.0 |
| 100 | < 5 | < 5 | < 5 | 53.7 ± 0.4 |
| 50 | 7.5 ± 2.6 | < 5 | < 5 | 47.2 ± 1.3 |
| 10 | < 5 | < 5 | < 5 | < 5 |
