# Supplementary material for: Chemical characterization and in vitro immunomodulatory effects of different extracts of moss Hedwigia ciliata (Hedw.) P. Beauv. from the Vršačke Planine Mts., Serbia
Source: PLoS One. 2021 Feb 11;16(2):e0246810. doi: 10.1371/journal.pone.0246810 (PMC7877662; doi:10.1371/journal.pone.0246810)
Supplement: S4 Table — The results are expressed as the mean ± SE relative to a non-treated control cells from a representative experiment of three independent experiments performed in quadruplicate. (DOCX) [file pone.0246810.s004.docx]

**S4 Table.**The antiproliferative and anti-inflammatoryeffects of the corresponding extracts (10 μg/mL), E1 (96 % ethanol), E2 (water:ethanol – 50:50, vol%), and E3 (ethyl acetate) on the MRC-5 cell line determined by MTT, NBT, and Griess test. The results are expressed as the mean ± SErelative to a non-treated control cells from a representative experimentofthree independent experiments performed in quadruplicate.

| **Assays** | **Samples** | | | |
| --- | --- | --- | --- | --- |
| **MTT test**  (Viability %) | **E1** | **E2** | **E3** | **Control** |
|  | 91.7 ± 1.3 | 90.3 ± 1.6 | 90.6 ± 1.1 | 100.0 |
| **NBT test**  (ROS index) | 1.3 ± 0.0 | 1.1 ± 0.1 | 1.0 ± 0.1 | 1.0 |
| **Griess assay**  (nitrite concentration μgmL^-1^ ) | 54.9± 1.4 | 54.5 ± 1.6 | 54.8 ± 0.3 | 53.0 |
